# Combining Review Text Content and Reviewer-Item Rating Matrix to Predict Review Rating

**DOI:** 10.1155/2016/5968705

**Published:** 2016-01-03

**Authors:** Bingkun Wang, Yongfeng Huang, Xing Li

**Affiliations:** Tsinghua National Laboratory for Information Science and Technology, Department of Electronic Engineering, Tsinghua University, Beijing 100084, China

## Abstract

E-commerce develops rapidly. Learning and taking good advantage of the myriad reviews from online customers has become crucial to the success in this game, which calls for increasingly more accuracy in sentiment classification of these reviews. Therefore the finer-grained review rating prediction is preferred over the rough binary sentiment classification. There are mainly two types of method in current review rating prediction. One includes methods based on review text content which focus almost exclusively on textual content and seldom relate to those reviewers and items remarked in other relevant reviews. The other one contains methods based on collaborative filtering which extract information from previous records in the reviewer-item rating matrix, however, ignoring review textual content. Here we proposed a framework for review rating prediction which shows the effective combination of the two. Then we further proposed three specific methods under this framework. Experiments on two movie review datasets demonstrate that our review rating prediction framework has better performance than those previous methods.

## 1. Introduction

Web 2.0 and e-commerce give rise to the explosion of online reviews. In turn, intelligently learning the sentiment propensity and opinions of these reviews is exactly the key to success in current wave of e-commerce. Binary classification, or positive-negative classification, of these reviews has been quite common but it gradually fails to meet the requirement of accuracy [1]. For instance, which item will be selected out of several items that all belong to the positive category is, therefore, hard to predict. However, even the nuances between review rates can lead to great difference in their volume of sales. The review rating prediction research of [2] shows that consumers are often willing to pay 20% to even 99% more money to buy a product of 5-star rate than that of 4-star rate.

There are mainly two types of methods of current review rating prediction (RRP). The first one based on review text content adopts the perspective of natural language process. Researchers transform review text into feature vectors and then employ a multiclass classifier or a regression model to predict review rates [3–7]. It simply ignores the relationship between the costumers and the items. The second one based on collaborative filtering (CF) focuses on the standpoints of recommenders [8, 9]. Researchers employ the *k*-nearest neighbor methods [10, 11] or matrix factorization methods [12–17] to extract information from previous reviewer-item rating matrix for review rating prediction. This type of method exploits no information from review text.

In order to include more information to accomplish finer-grained review rating prediction, we proposed a framework combining review text content with previous reviewer-item rating matrix. After that, we contrived three specific methods under this framework. Then we did some experiments on two movie review datasets to examine the efficiency of our framework and three methods. And the result shows that our methods under this framework by and large refine the performance of RRP and generate desirable results.

The outline of the paper is as follows.  Section 2 introduces those related researches on RRP. RRP based on review text content and RRP based on CF are described, respectively, in Section 3.  Section 4 presents our framework and the three methods under it. Experimental results on two movie review datasets are reported in Section 5. Finally, Section 6 concludes the paper and points out our future research direction.

## 2. Related Work

The research of sentiment classification is mainly divided into two aspects [18, 19]: one is positive-negative binary classification; the other is the fine-grained RRP. As to binary classification of reviews, some of the creative methods for achieving this have been discussed in [20, 21]. In RRP, there are many research results proposed [3–17], but accuracy of RRP still cannot meet the real demands at present.

At present, there are mainly two ways of fulfilling finer-grained RRP. One includes methods based on review text content (MBRTC), which mines information from review text content by discerning and quantifying a variety of text features and then employs the regression model to predict review rating [7, 22]. For example, Qu et al. [7] consider RRP as a feature engineering problem, and extracted various features, such as words, patterns, syntactic structures, and semantic topics, from the review text to improve the performance. Wang et al. [22] proposed a type of methods based on the content of review and weighting strong social relation of reviewers to predict review rating. To be specific, they predict review rating by incorporating the character of reviewer's social relations, as regularization constraints, into content-based methods. The main problem of MBRTC is that it mainly uses the review text content information and does not refer to reviewer-items rating matrix information.

There are also some researches based on review text content taking into account characteristics of the items or the reviewers [5, 23]. Wang et al. [23] noticed that the score that a piece of review relates to cannot be fully determined by the review content itself, since review content is not an absolute metric of sentiment propensity. A tough reviewer may use tough words for all items, even items that he rates high. Different items have to meet different basic requirements. Simply analyzing the review content is not enough. Li et al. [5] proposed a method of incorporating reviewer and item information into review text content. They consider the personal characteristics of the reviewers when mining reviews content and use tensor factorization techniques to learn parameter of regression model and predict reviews rating. This method only considers the effect of reviewer and item on review text content and then uses review text content to predict review rating, which is indeed a method based on review text content.

The other one contains methods based on CF (MBCF). Those methods can be further divided into two categories. The first one dictates people to do similarity calculation to find the *k*-nearest neighbor reviewers or items to do prediction [11, 12]. The second one requires people to use the latent factor model to fulfill the matrix factorization. Several low-dimensional matrix factorization techniques are presented in [24–26]. Koren et al. [12–14] proposed several enhanced matrix factorization methods which can generate promising results by applying heterogeneous information to object functions. Koren [12] built a combined model by merging the matrix factorization and neighborhood models and improved accuracy of recommendation by extending the models to exploit both explicit and implicit feedback by the users. Koren [14] proposed a method to model the time changing behavior throughout the life span of the data and improved the performance of recommendation. In [27], researchers extended the matrix factorization objective function with the social network information of reviewers. In [28], Shi et al. proposed a context-aware movie recommendation algorithm based on joint matrix factorization (JMF). They jointly factorize the user-item matrix containing general movie ratings and other contextual movie similarity matrixes to integrate contextual information into the recommendation process.

Up to now, some researches combining ratings and text reviews have been applied to recommend system [29, 30]. For example, Cremonesi et al. proposed a hotel recommender algorithm (Interleave), which provides recommendations based on the text reviews and ratings [29]. Levi et al. proposed a recommender system that combines reviews and ratings to recommend hotels [30]. But as far as we know, the methods based on combining ratings and text reviews have not been applied in review rating prediction. Different from the existing methods focusing on recommend system, we focused on review rating prediction and proposed a general framework and three special methods based on review text content and reviewer-item matrix.

Different from the above methods, we propose a framework combining MBRTC and MBCF to include more information to improve the accuracy of prediction. We also present three specific methods under this framework. Finally, the experiment results verify effectiveness of our proposed framework and methods.

## 3. RRP Based on Review Text Content and RRP Based on Reviewer-Item Rating Matrix

For an online review site with *N* items *I* = {*i*_1_, *i*_2_,…, *i*_*N*_}, *T* reviews *R* = {*r*_1_, *r*_2_,…, *r*_*T*_} about the *N* items, *M* reviewers *A* = {*a*_1_, *a*_2_,…, *a*_*M*_} who have written the *T* reviews, and *T*1 review ratings *V*1 = {*v*_1_, *v*_2_,…, *v*_*T*1_} which are corresponding to *T*1 reviews *R*1 = {*r*_1_, *r*_2_,…, *r*_*T*1_}, our goal is to predict rating of each review in *R*2 = {*r*_*T*1_, *r*_*T*1+1_,…, *r*_*T*_}. In this section, we introduce two existing types of methods in review rating prediction. One is based on review text content; the other one is based on collaborative filtering.

### 3.1. RRP Based on Review Text Content

Review text content is a very important information source for RRP. Current review text content-based RRP methods mainly use vector space model (VSM) to express review text content and then use linear regression model to predict review rating. To be specific, there are four steps to take. Firstly, online review text content, which includes segmentations of terms, part-of-speech tagging, and frequency statistics, should be preprocessed. Secondly, regarding words, phrases, and *n*-gram as features, people employ some feature selection methods to choose features that can perfectly express the review text content to compose the feature set *F* = {*f*_1_, *f*_2_,…, *f*_*L*_}. Thirdly, each online review *r*_*i*_ in *R* = {*r*_1_, *r*_2_,…, *r*_*T*_} is expressed as an *L*-dimensional vector which is exactly an instantiated value of *F*. Fourthly, the linear regression model dealing with those vectors of reviews is adopted to predict review rating. The linear regression model is described in

(1) $$\begin{array}{c} {\hat{v}}_{i}=w^{T}r_{i}. \end{array}$$

To work out the parameter vector *w*, given training datasets with *R*1 = {*r*_1_, *r*_2_,…, *r*_*T*1_} and *V*1 = {*v*_1_, *v*_2_,…, *v*_*T*1_} available, least squares error loss is used to minimize the objective function:

(2) $$\begin{array}{c} \underset{w}{\min}\underset{\text{trainsets}}{\sum }{\left(\;v_{i}-w^{T}r_{i}\;\right)}^{2}+\lambda {\left\|\;w\;\right\|}^{2}. \end{array}$$

Here, ‖*w*‖^2^, the regularization term of parameter vector *w*, is employed to avoid overfitting; *λ* is the regularization coefficient. To estimate the parameters *w*, a simple stochastic gradient descent algorithm is adopted to solve the optimization problem. For each observed rating *v*_*i*_ ∈ *V*1 = {*v*_1_, *v*_2_,…, *v*_*T*1_}, we refer to the following updating rules to learn the parameters *w*:

(3) $$\begin{array}{c} w⟵w+\eta \left(\;{ɛ}_{i}r_{i}-\lambda w\;\right). \end{array}$$

Here *ɛ*_*i*_ = *v*_*i*_ − *w* ^*T*^ *r*_*i*_, and *η* are the learning rates. After getting *w*, given *R*2 = {*r*_*T*1_, *r*_*T*1+1_,…, *r*_*T*_}, we can apply ${\hat{v}}_{i}=w^{T}r_{i}$ to predict the review rating of each review in *R*2.

### 3.2. RRP Based on Collaborative Filtering

RRP now plays an essential role in recommend system. At present, RRP for recommend system mainly based on collaborative filtering involves two methods. One uses the *k*-nearest neighbor to predict and estimate the current object. The other one uses matrix factorization.

#### 3.2.1. RRP Based on the *k*-Nearest Neighbor Model

RRP based on the *k*-nearest neighbor model includes the reviewer-based method and the item-based method. With the reviewer-item rating matrix available, a typical reviewer-based approach is to predict a reviewer's rating on a target item by aggregating the previous ratings on it from *k*-nearest reviewers. We can consequently formulate the predicted rating on item *i* from reviewer *x* as follows:

(4) $$\begin{array}{c} {\hat{v}}_{xi}=\underset{C}{\sum }s_{xx^{\prime }}v_{x^{\prime }i}. \end{array}$$

Here, *C* is the set of *k* nearest neighbor of reviewer *x* and *s*_*xx*′_ represents the similarity between reviewer *x* and reviewer *x*′; *v*_*x*′*i*_ is review rating from reviewer *x*′ on item *i*.

To get the parameter *s*_*xx*′_, given training datasets with *R*1 = {*r*_1_, *r*_2_,…, *r*_*T*1_} and *V*1 = {*v*_1_, *v*_2_,…, *v*_*T*1_} available, we have to solve the optimization problem, that is, to minimize the square error loss function below:

(5) $$\begin{array}{c} \underset{s_{xx^{\prime }}}{\min}\underset{\text{trainsets}}{\sum }{\left(\;v_{xi}-\sum_{C}s_{xx^{\prime }}v_{x^{\prime }i}\;\right)}^{2}+\lambda {\left\|\;s_{xx^{\prime }}\;\right\|}^{2}. \end{array}$$

Here ‖*s*_*xx*′_‖^2^ is a regularization term of parameter *s*_*xx*′_ aimed at avoiding overfitting and *λ* is regularization coefficient. Then, a simple stochastic gradient descent algorithm is adopted to solve the optimization problem. For each observed rating *v*_*xi*_ ∈ *V*1 = {*v*_1_, *v*_2_,…, *v*_*T*1_}, we refer to the following updating rules to learn the parameters *s*_*xx*′_:

(6) $$\begin{array}{c} s_{x}⟵s_{x}+\eta \left(\;{ɛ}_{xi}\underset{C}{\sum }v_{x^{\prime }i}-\lambda s_{x}\;\right). \end{array}$$

Here, *ɛ*_*xi*_ = *v*_*xi*_ − ∑_*C*_ *s*_*xx*′_ *v*_*x*′*i*_ and *η* are the learning rates. After getting *s*_*xx*′_, we can apply ${\hat{v}}_{xi}=\sum_{C}s_{xx^{\prime }}v_{x^{\prime }i}$ to predict the review rating.

#### 3.2.2. RRP Based on Matrix Factorization

Matrix factorization (MF) is one of the most popular methods in recommend system. The kernel of MF is to find a small number of latent features that might relate to the preferences of reviewer and use them to match observed ratings. A typical model associates each reviewer *x* with a vector of reviewer factors and each item *i* with a vector of item factors. The prediction is done through an inner product which is described by

(7) $$\begin{array}{c} {\hat{v}}_{xi}=p_{x}q_{i}^{T}. \end{array}$$

In order to compute the two parameters *p*_*x*_ and *q*_*i*_, we follow the least squares error loss principle to minimize the objective function:

(8) $$\begin{array}{c} \underset{p_{x},q_{i}}{\min}\underset{\text{trainsets}}{\sum }{\left(\;v_{xi}-p_{x}q_{i}^{T}\;\right)}^{2}+\lambda \left(\;{\left\|\;p_{x}\;\right\|}^{2}+{\left\|\;q_{i}\;\right\|}^{2}\;\right). \end{array}$$

Here ‖*p*_*x*_‖^2^ and ‖*q*_*i*_‖^2^ are the regularization terms of parameters *p*_*x*_ and *q*_*i*_ serving to avoid overfitting; *λ* is the regularization coefficient. In order to estimate the parameters *p*_*x*_ and *q*_*i*_, a simple gradient descent algorithm was successfully applied to solve the optimization problem. For each observed rating *v*_*xi*_ ∈ *V*1 = {*v*_1_, *v*_2_,…, *v*_*T*1_}, we used the following updating rules to acquire the parameters *p*_*x*_ and *q*_*i*_:

(9) px⟵px+ηɛxiqi−λpx,qi⟵qi+ηɛxipx−λqi.

Here, *ɛ*_*xi*_ = *v*_*xi*_ − *p*_*x*_ *q*_*i*_^*T*^ and *η* are the learning rates. After getting *p*_*x*_ and *q*_*i*_, we can apply ${\hat{v}}_{xi}=p_{x}q_{i}^{T}$ to predict review rating.

## 4. RRP by Combining Review Text Content and Reviewer-Item Rating Matrix

### 4.1. Problem Description

In order to illustrate the problem we study in this paper, a toy example about reviewers, items, review text content, and review rating is shown in Table 1. From the toy example, we can get three types of information: the user-item rating matrix, review text content with corresponding rating, and review text content without corresponding rating. The problem we study is how to effectively predict missing rating of each review in user-item rating matrix. In this section, we propose a new RRP framework combining reviewer-item rating matrix (RIRM) with review text content (RTC). That is, we want to find a function *f* : (RIRM, RTC)→(rating).

### 4.2. General Framework

There are mainly two types of methods in existing RRP. One includes the methods based on review text content, which can be described as a function *f*1 : (RTC)→(rating). The other one contains the methods based on collaborative filtering, which can be described as a function *f*2 : (RIRM)→(rating).

The above two types of methods use either review text content or reviewer-item rating matrix, not having made full use of all the information available. Therefore, we proposed a RRP framework combining review text content and reviewer-item rating matrix. The framework is described in

(10) $$\begin{array}{c} f\left(\;\text{RIRM},\text{RTC}\;\right)=\left(\;1-\alpha \;\right)f1\left(\;\text{RTC}\;\right)+\alpha f2\left(\;\text{RIRM}\;\right). \end{array}$$

According to the general framework, we proposed three specific special RRP methods. In order to improve the performance of the three special RRP methods, we contrived a way to compute the parameters in the three special RRP methods.

### 4.3. Three Special RRP Methods

#### 4.3.1. RRP Combining Linear Regression Model and *k*-Nearest Neighbor

We choose the linear regression model as *f*1(RTC) and *k*-nearest neighbor to form *f*2(RIRM). The special methods are described in

(11) $$\begin{array}{c} {\hat{v}}_{xi}=\left(\;1-\alpha \;\right)wr_{xi}+\alpha \underset{x^{\prime }}{\sum }s_{xx^{\prime }}v_{x^{\prime }i}. \end{array}$$

In order to get optimum parameters *α*, *w*, *s*_*xx*′_, given training datasets with *M* reviewers *A* = {*a*_1_, *a*_2_,…, *a*_*M*_} who have written *T*1 review ratings *V*1 = {*v*_1_, *v*_2_,…, *v*_*T*1_} which are corresponding to *T*1 reviews *R*1 = {*r*_1_, *r*_2_,…, *r*_*T*1_}, we follow the least squares error loss principle to minimize the objective function:

(12) fw,sxx′=λw2+sxx′2+minw,sxx′⁡∑trainsetsvxi−1−αwrxi−α∑x′sxx′vx′i2.

Here *w* and *s*_*xx*′_ are regularization terms of those parameters aimed at avoiding overfitting; *λ* is a regularization coefficient. In order to estimate the parameters *α*, *w*, and *s*_*xx*′_, we firstly traverse *α* from 0 to 1. Secondly, for each fixed *α* in training dataset, we then adopt a simple stochastic gradient descent algorithm to solve the optimization problem. For each observed rating *v*_*xi*_ ∈ *V*1 = {*v*_1_, *v*_2_,…, *v*_*T*1_}, we refer to the following updating rules:

(13) w⟵w+η1−αɛxirxi−λw,sx⟵sx+ηαɛxi∑x′vx′i−λsx.

Here *ɛ*_*xi*_ = *v*_*xi*_ − (1 − *α*)*wr*_*xi*_ − *α*∑_*x*′_ *s*_*xx*′_ *v*_*x*′*i*_, and *η* are the learning rates. After getting *α*, *w*, and *s*_*xx*′_, given *R*2 = {*r*_*T*1_, *r*_*T*1+1_,…, *r*_*T*_}, we can apply ${\hat{v}}_{xi}=(1-\alpha )wr_{xi}+\alpha \sum_{x^{\prime }}s_{xx^{\prime }}v_{x^{\prime }i}$ to predict review rating of each review in *R*2.

#### 4.3.2. RRP Combining Linear Regression Model and Matrix Factorization

Here, we choose linear regression model as *f*1(RTC) and Matrix Factorization as *f*2(RIRM). The special methods are described in

(14) $$\begin{array}{c} {\hat{v}}_{xi}=\left(\;1-\beta \;\right)wr_{xi}+\beta p_{x}q_{i}^{T}. \end{array}$$

To acquire optimum parameters *β*, *w*, *p*_*x*_, and *q*_*i*_, given training datasets, we minimize the objective function according to the least square error loss principle:

(15) fw,px,qi=λw2+px2+qi2+minw,px,qi⁡∑trainsetsvxi−1−βwrxi−βpxqiT2.

Here *w*, *p*_*x*_, and *q*_*i*_ are regularization terms of those parameters serving to avoid overfitting; *α* is a regularization coefficient. In order to estimate the parameters *β*, *w*, *p*_*x*_, and *q*_*i*_, we firstly traverse *β* from 0 to 1. Secondly, for each fixed *β* in training dataset, we adopt a simple stochastic gradient descent algorithm to solve the optimization problem. For each observed rating *v*_*xi*_ ∈ *V*1 = {*v*_1_, *v*_2_,…, *v*_*T*1_}, we refer to the following updating rules:

(16) w⟵w+η1−βɛxirxi−λw,px⟵px+ηβɛxiqi−λpx,qi⟵qi+ηβɛxipx−λqi.

Here *ɛ*_*xi*_ = *v*_*xi*_ − (1 − *β*)*wr*_*xi*_ − *βp*_*x*_ *q*_*i*_^*T*^, and *η* are the learning rates. After learning *β*, *w*, *p*_*x*_, and *q*_*i*_, for *R*2 = {*r*_*T*1_, *r*_*T*1+1_,…, *r*_*T*_}, we can apply ${\hat{v}}_{xi}=(1-\beta )wr_{xi}+\beta p_{x}q_{i}^{T}$ to predict the review ratings of it.

#### 4.3.3. RRP by Combining Linear Regression Model, the *k*-Nearest Neighbor, and Matrix Factorization

In RRP framework, we choose linear regression model as *f*1(RTC) and *k*-nearest neighbor and Matrix Factorization as *f*2(RIRM). The special methods are described in

(17) $$\begin{array}{c} {\hat{v}}_{xi}=\left(\;1-\alpha -\beta \;\right)wr_{xi}+\alpha \underset{x^{\prime }}{\sum }s_{xx^{\prime }}v_{x^{\prime }i}+\beta p_{x}q_{i}^{T}. \end{array}$$

In order to get optimum parameters *α*, *β*, *w*, *s*_*xx*′_, *p*_*x*_, and *q*_*i*_, given training datasets available, we follow the least square error loss principle to minimize the objective function:

(18) fw,sxx′,px,qi=λw2+sxx′2+px2+qi2+minw,sxx′,px,qi⁡∑trainsetsvxi−1−α−βwrxi−α∑x′sxx′vx′i−βpxqiT2.

Here *w*, *s*_*xx*′_, *p*_*x*_, and *q*_*i*_ are regularization terms of those parameters serving to avoid overfitting; *λ* is a regularization coefficient. In order to estimate the parameters *α*, *β*, *w*, *s*_*xx*′_, *p*_*x*_, and *q*_*i*_, we firstly traverse *α* from 0 to 1. Secondly, for each fixed *α* in training datasets, we traverse *β* from 0 to 1. Thirdly, for each fixed *α* and *β* in training datasets, we adopt a simple stochastic gradient descent algorithm to solve the optimization problem. For each observed rating *v*_*xi*_ ∈ *V*1 = {*v*_1_, *v*_2_,…, *v*_*T*1_}, we refer to the following updating rules:

(19) w⟵w+η1−α−βɛxirxi−λw,sx⟵sx+ηαɛxi∑x′vx′i−λsx,px⟵px+ηβɛxiqi−λpx,qi⟵qi+ηβɛxipx−λqi.

Here *ɛ*_*xi*_ = *v*_*xi*_ − (1 − *α* − *β*)*wr*_*xi*_ − *α*∑_*x*′_ *s*_*xx*′_ *v*_*x*′*i*_ − *βp*_*x*_ *q*_*i*_^*T*^ and *η* are the learning rates. After learning *α*, *β*, *w*, *s*_*xx*′_, *p*_*x*_, and *q*_*i*_, for *R*2 = {*r*_*T*1_, *r*_*T*1+1_,…, *r*_*T*_}, we can apply ${\hat{v}}_{xi}=\left(1-\alpha -\beta \right)wr_{xi}+\alpha \sum_{x^{\prime }}s_{xx^{\prime }}v_{x^{\prime }i}+\beta p_{x}q_{i}^{T}$ to predict the review ratings of it.

## 5. Evaluations

### 5.1. Datasets and Experimental Setup

In order to verify the performance of our proposed framework and methods, we performed several experiments on two datasets from the popular review site http://www.douban.com/. This website is a reviewer-opinion website where reviewers can read and write reviews on movies, music, and books and mark a rating from 1 star to 5 stars. We downloaded information of both movies and reviewers, movie reviews, and their ratings through the API of http://movie.douban.com/ community. The rough description of the two datasets is shown in Table 2.

In Table 2, reviewer-item rating matrix density (RIRMD) is calculated by

(20) $$\begin{array}{c} \text{RIRMD}=\frac{\text{Number }\left(\text{review}\right)}{\text{Number }\left(\text{reviewer}\right)\times \text{Number }\left(\text{item}\right)}. \end{array}$$

To evaluate the overall performance of our framework and methods, we divide datasets into 10 parts at random. We do the experiment with taking 80% reviews for training and the remaining 20% reviews for test. We compare our framework and methods with methods based either on review text content or on reviewer-item rating matrix through experiments on two different datasets. The six different methods are abbreviated as follows:

 MBLR: methods based on linear regression model.
 MBKNN: methods based on *k*-nearest neighbor.
 MBMF: methods based on matrix factorization.
 MBLR + MBKKN: our method combining linear regression model and *k*-nearest neighbor.
 MBLR + MBMF: our method combining linear regression model and matrix factorization.
 MBLR + MBKNN + MBMF: our method combining linear regression model, *k*-nearest neighbor, and matrix factorization.

There are mainly two factors influencing RRP in our framework. One is review text content information. The second is review-item matrix information. We did four experiments on two datasets to answer the following four questions:

(1) How to set parameters *α* and *β*, and what are the effects of different parameters *α* and *β* on MAE and RMSE of RRP?
(2) Can our framework and methods decrease MAE and RMSE of RRP?
(3) Is the algorithm complexity of our methods higher than the three single methods?
(4) What is the relationship between RIRMD and MAE and RMSE of RRP?

We use root mean square error (RMSE) and mean absolute error (MAE) as metric to evaluate performance of different RRP methods. RMSE and MAE are computed by

(21) MAE=∑testsetsv^xi−vxiNtotal,RMSE=∑testsetsv^xi−vxi2Ntotal,

where ${\hat{v}}_{xi}$ is the predicting rating by all kinds of methods, *v*_*xi*_ is the rating we have got from test datasets, and *N*_total_ is the number of reviews in test datasets.

### 5.2. Performance Evaluation

#### 5.2.1. Setting Parameters *α* and *β* in Our Frameworks

**Figure 1:**
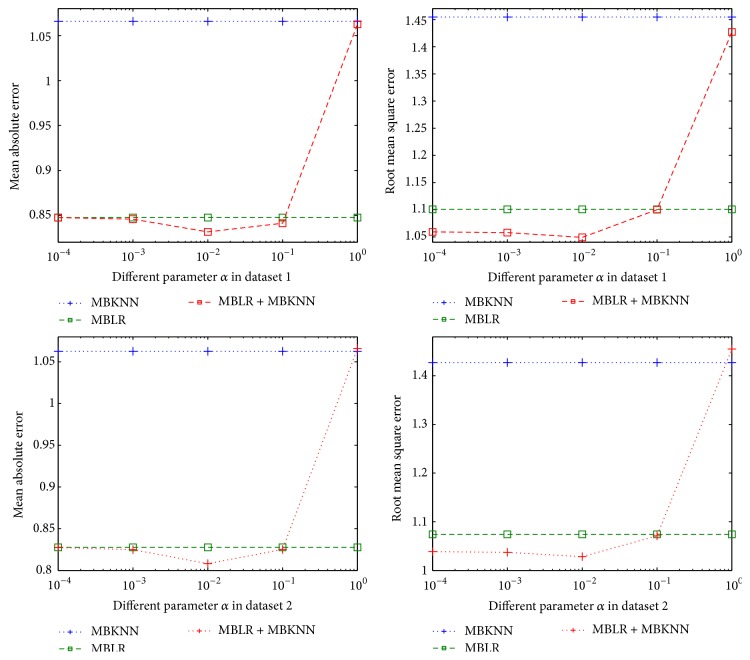
MAE and RMSE of MBLR + MBKKN changes with parameter *α*.

**Figure 2:**
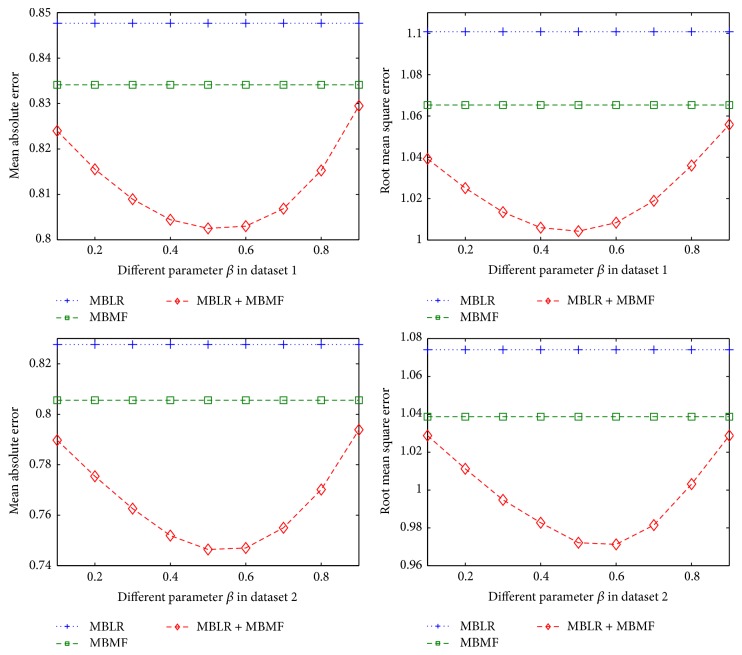
MAE and RMSE of MBLR + MBMF changes with parameter *β*.

**Figure 3:**
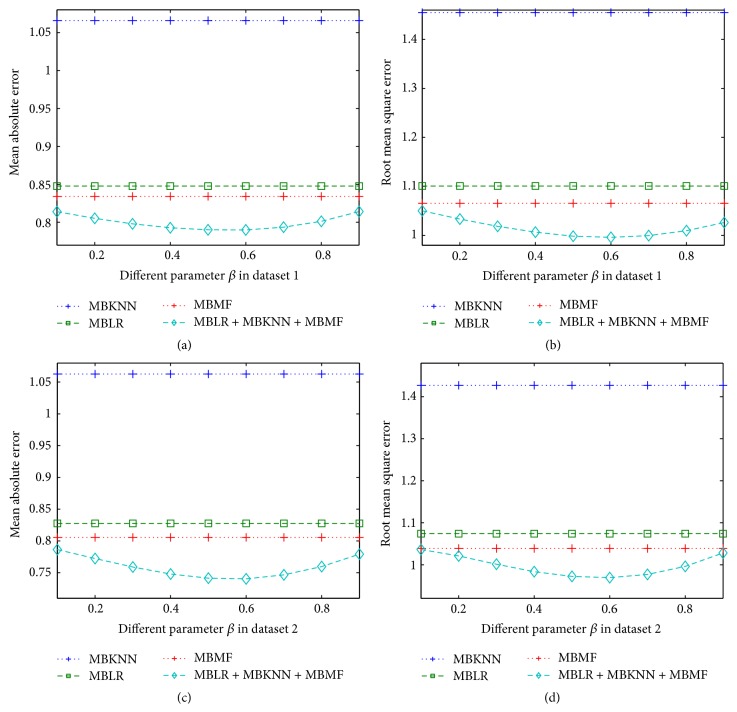
MAE and RMSE of MBLR + MBKNN + MBMF changes with parameter *β*.

In our framework, there are two parameters that have to be set, namely, *α* and *β*. We perform 10-fold cross validation in training datasets to get the optimum value of parameters *α* and *β*.  Figure 1 shows how MAE and RMSE of MBLR + MBKKN change with parameter *α* in two different training datasets.  Figure 2 shows how MAE and RMSE of MBLR + MBMF change with parameter *β* in two different training datasets.  Figure 3 shows how MAE and RMSE of MBLR + MBKNN + MBMF change with parameter *β* in two different training datasets.

From Figures 1, 2, and 3, we can see that MAE and RMSE of RRP in our methods change with different parameters *α* and *β*, which proved that parameters *α* and *β* play very important roles in our methods. So, we need to find optimum parameters *α* and *β* in our methods to improve the performance of RRP.

In Figure 1, when parameter *α* = 0.01, MAE and RMSE of MBLR + MBKNN are the lowest. The reason is that reviewer-items rating matrix of dataset 1 and dataset 2 is very sparse. It is well known that *k*-nearest neighbor method based on CF has poor performance on very sparse datasets. At the same time, linear regression model based on review text content has better performance than *k*-nearest neighbor method based on CF in dataset 1 and dataset 2.

In Figure 2, when parameter *β* is set between 0.5 and 0.6, MAE and RMSE of MBLR + MBMF are the lowest. The reason is that MF method based on CF has slightly better performance than linear regression model based on review text content.

In Figure 3, we fix *α* = 0.01. When parameter *β* is chosen between 0.5 and 0.6, MAE and RMSE of MBLR + MBKNN + MBMF are the lowest. The reason is that both MF method based on CF and linear regression model based on review text content have better performance than *k*-nearest neighbor method based on CF when reviewer-items rating matrix of datasets is very sparse. At the same time, MF method based on CF has slightly better performance than linear regression model based on review text content.

Therefore, we set those parameters of our three methods according to the above results. The special parameters of the three different methods are shown in Table 3.

The MAE and RMSE of the three single methods are changed in dataset 1 and dataset 2, but the relative performance of the three single methods is basically unchanged in dataset 1 and dataset 2. For example, the gap between RMSE of the MBKNN and MBLR in dataset 1 and dataset 2 is basically identical (0.353). The gap between RMSE of the MBLR and MBMF in dataset 1 and dataset 2 is basically identical (0.035). So when we combining the three different single methods, we can preliminary choose parameter according to the relative performance of the three different single methods. The gap between RMSE of MBLR and MBMF is 10 times bigger than the gap between RMSE of MBKNN and MBLR.

From Figures 1, 2, and 3, we can see that the performance of our proposed methods is continuously better than single methods when parameter is chosen according to the relative performance of the three different single methods. At the same time, when parameter changed in range of the relative performance of the three different single methods, our proposed methods are continuously better than single methods. For example, we can see that parameter beta changes between 0.1 and 0.9; our methods obtain always better performance than MBKNN, MBLR, and MBMF in Figures 2 and 3. So, in real world applications, our methods, which are obtained by training with one dataset, can be applied to many different ones.

#### 5.2.2. Effect of Different Methods to MAE and RMSE of RRP

In order to verify the performance of our proposed framework and methods, we compare our methods with three baseline methods in two different datasets. The experience results of those six methods on two datasets are presented in Table 4.

In both two datasets, MAE and RMSE of MBLR + MBKNN are lower than that of MBLR and MBKNN; MAE and RMSE of MBLR + MBMF are lower than that of MBLR and MBMF; MAE and RMSE of MBLR + MBKNN + MBMF are lower than that of MBLR, MBKNN, and MBMF. Experimental results prove that combining text content information and review-item matrix information can enhance the performance of RRP. This is because both of the two different information sources are not redundant and can therefore play their own role. When we combine the two types of information: text content information and review-item matrix information, performance of RRP is improved in a certain extent.

For example, MBLR + MBKNN decreases 4.7% ((1.100798 − 1.049105)/1.100798) RMSE compared to MBLR in dataset 1. MBLR + MBMF decreases 5.73% ((1.065305 − 1.004237)/1.065305) RMSE compared to MBMF in dataset 1. MBLR + MBKNN + MBMF decreases 6.51% ((1.065305 − 0.995946)/1.065305) RMSE compared to MBMF in dataset 1. Although MBLR + MBKNN + MBMF only decreases 0.78% RMSE compared to MBLR + MBMF, both the two methods are our proposed methods. Compared to the three single methods, our proposed methods all decrease the RMSE of RRP and improve the performance of RRP.

#### 5.2.3. Analyzing Complexity of Different Methods

Firstly, we compute the algorithm complexity of three simple approaches in Section 3. When computing the parameter of the objective function in the three methods, a simple stochastic gradient descent algorithm is adopted. According to the complexity of the stochastic gradient descent algorithm, we can get the algorithm complexity of the three simple approaches in Section 3. The algorithm complexity of those three methods on two datasets is presented in Table 4.

Then we compute the algorithm complexity of three special RRP approaches in Section 4. Similar to Section 3, when computing the parameter of the three special RRP methods, we also adopt a simple stochastic gradient descent algorithm to solve the optimization problem of minimizing the objective function. According to the complexity of the stochastic gradient descent algorithm and the computing course of the three special RRP methods, we can get the algorithm complexity of the three specific special RRP methods. The algorithm complexity of those three methods on two datasets is presented in Table 4.

From Table 4, we can see that the algorithm complexity of our proposed three methods in Section 4 is one order of magnitude with the algorithm complexity of the three simple methods in Section 3. When our methods obtain better results than the individual methods, the cost of our method is acceptable.

#### 5.2.4. The Relations between Performance of RRP and RIRMD

**Figure 4:**
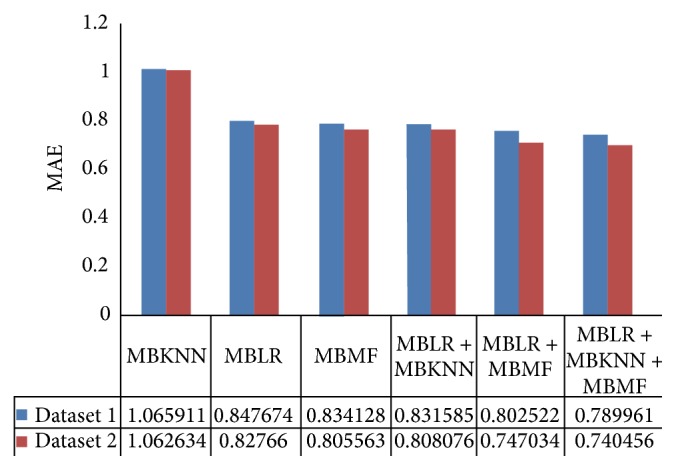
Relations between MAE of RRP and RIRMD.

**Figure 5:**
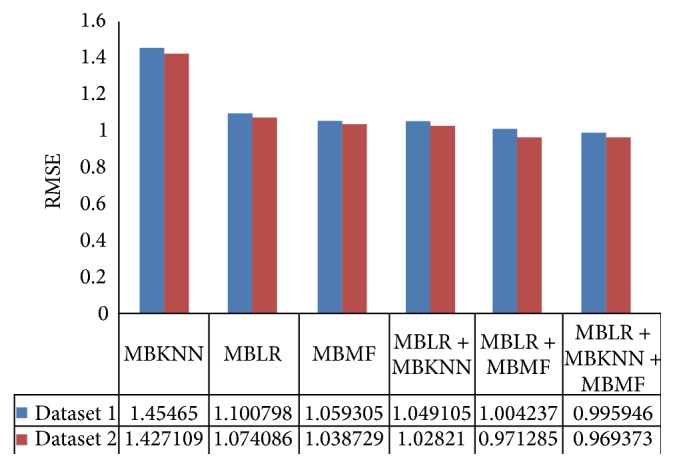
Relations between RMSE of RRP and RIRMD.

In order to evaluate the effect that the reviewer-item rating matrix density may have on review rating prediction, we experimented on two movie review datasets which are different in RIRMD. The experimental results are shown in Figures 4 and 5.

RIRMD of dataset 2 is denser than dataset 1. From Figures 4 and 5, we can see that MAE and RMSE values of dataset 2 are always lower than that of dataset 1 in our methods, which suggests that higher RIRMD always brings about lower MAE and RMSE. This is because higher RIRMD means more sufficient reviewer-item matrix information. When we combine more sufficient reviewer-item matrix information with review text content, we can get more accurate result statistically.

## 6. Conclusion

In this paper, we studied the previous methods of RRP and proposed a RRP framework combining review text content and reviewer-item rating matrix to make full use of all information sources to improve the performance of prediction. Based on RRP framework, we further contrived three specific RRP methods. Our methods have significantly enhanced the performance of RRP, compared to methods based solely on review text content or collaborative filtering. In the future, we will further experiment on frameworks combining review text content and reviewer-item matrix while employing the probability graph models.

## Figures and Tables

**Table 1:** A toy example.

Reviewers	Item 1	Item 2	Item 3	Item 4
Reviewer 1	Review text content, 5	Review text content, ?	Review text content, 4	
Reviewer 2		Review text content, 3		Review text content, ?
Reviewer 3	Review text content, 4		Review text content, ?	
Reviewer 4	Review text content, ?	Review text content, 2		Review text content, ?
Reviewer 5	Review text content, ?		Review text content, 5	Review text content, 3

**Table 2:** Douban movie reviews datasets.

Datasets	Reviewer	Review	Item	Matrix density
Dataset 1	1476	22593	3041	0.005034
Dataset 2	1079	13858	2087	0.006154

**Table 3:** Parameters *α* and *β* of three methods.

Methods	MBLR + MBKNN	MBLR + MBMF	MBLR + MBKNN + MBMF
Parameter	*α* = 0.01	*β* = 0.55	*α* = 0.01, *β* = 0.55

**Table 4:** Douban movie reviews datasets.

Datasets	Metrics	MBKNN	MBLR	MBMF	MBLR + MBKNN	MBLR + MBMF	MBLR + MBKNN + MBMF
Dataset 1	MAE	1.065911	0.847674	0.834128	0.831585	0.802522	0.789961
Dataset 1	RMSE	1.454650	1.100798	1.065305	1.049105	1.004237	0.995946
Dataset 1	Complexity	*O*(*k*)	*O*(*L*)	*O*(2*S*)	*O*(*L* + *k*)	*O*(*L* + 2*S*)	*O*(*L* + *k* + 2*S*)

Dataset 2	MAE	1.062634	0.827660	0.805563	0.808076	0.747034	0.740456
Dataset 2	RMSE	1.427109	1.074086	1.038729	1.028210	0.971285	0.969373
Dataset 2	Complexity	*O*(*k*)	*O*(*L*)	*O*(2*S*)	*O*(*L* + *k*)	*O*(*L* + 2*S*)	*O*(*L* + *k* + 2*S*)

*L*: dimension of VSM; *k*: number of reviewer's nearest neighbor; *S*: number of latent factor in ratings matrix.
